# Validation of an enzyme-linked immunosorbent assay for the quantification of human IgG directed against the repeat region of the circumsporozoite protein of the parasite *Plasmodium falciparum*

**DOI:** 10.1186/1475-2875-11-384

**Published:** 2012-11-22

**Authors:** Frederic Clement, Vincent Dewar, Eva Van Braeckel, Isabelle Desombere, Marianne Dewerchin, Christine Swysen, Marie-Ange Demoitié, Erik Jongert, Joe Cohen, Geert Leroux-Roels, Pierre Cambron

**Affiliations:** 1 Center for Vaccinology, Ghent University, Ghent, Belgium; 2 GlaxoSmithKline Vaccines, Rixensart, Rue de l’Institut 89, B-1330, Rixensart, Belgium

**Keywords:** Malaria, *Plasmodium falciparum*, Circumsporozoite protein, Enzyme-linked immunosorbent assay, R32LR, Validation

## Abstract

**Background:**

Several pre-erythrocytic malaria vaccines based on the circumsporozoite protein (CSP) antigen of *Plasmodium falciparum* are in clinical development. Vaccine immunogenicity is commonly evaluated by the determination of anti-CSP antibody levels using IgG-based assays, but no standard assay is available to allow comparison of the different vaccines.

**Methods:**

The validation of an anti-CSP repeat region enzyme-linked immunosorbent assay (ELISA) is described. This assay is based on the binding of serum antibodies to R32LR, a recombinant protein composed of the repeat region of *P. falciparum* CSP. In addition to the original recombinant R32LR, an easy to purify recombinant His-tagged R32LR protein has been constructed to be used as solid phase antigen in the assay. Also, hybridoma cell lines have been generated producing human anti-R32LR monoclonal antibodies to be used as a potential inexhaustible source of anti-CSP repeats standard, instead of a reference serum.

**Results:**

The anti-CSP repeats ELISA was shown to be robust, specific and linear within the analytical range, and adequately fulfilled all validation criteria as defined in the ICH guidelines. Furthermore, the coefficient of variation for repeatability and intermediate precision did not exceed 23%. Non-interference was demonstrated for R32LR-binding sera, and the assay was shown to be stable over time.

**Conclusions:**

This ELISA, specific for antibodies directed against the CSP repeat region, can be used as a standard assay for the determination of humoral immunogenicity in the development of any CSP-based *P. falciparum* malaria vaccine.

## Background

Malaria, due to infection by the protozoan genus *Plasmodium*, is a major cause of morbidity and mortality worldwide, being responsible for 655,000 [1] to 1,238,000 [2] deaths a year in 2010, mostly children in sub-Saharan Africa. In addition to the existing prevention and control measures, malaria vaccines are considered the most promising approach for the prevention of malaria disease and death, and the World Health Organization has referenced up to 23 malaria vaccine projects under clinical development [3]. The RTS,S vaccine candidate, based on circumsporozoite protein (CSP) sequences of *Plasmodium falciparum* (for review, see [4]), is the most advanced among these and is currently engaged in a large scale phase III clinical trial program [5,6].

Specific anti-CSP IgG levels are a relevant parameter in CSP-based malaria vaccine projects, as there is evidence from preclinical models that anti-CSP antibodies contribute to protection against malaria during the pre-erythrocytic stage of the disease [7-11]. In line with this, an association between anti-CSP antibody levels and protection against *P. falciparum* infection or clinical malaria disease has been observed in humans participating to RTS,S-based vaccine trials [12-20]. It has been suggested that opsonization of the sporozoites by anti-CSP antibodies is at least one of the mechanisms inducing protective immunity [21].

The Asn-Ala-Asn-Pro (NANP) sequence is the major B-cell epitope of the *P.falciparum* CSP [22], and NANP-based peptides have already been widely used in immunoassays aimed at detecting anti-CSP antibodies [23-26]. Recombinant proteins composed of 15 NANP repeats and an Asn-Val-Asp-Pro (NVDP) oligopeptide, NVDP(NANP)_15_, 30 NANP repeats, [NVDP(NANP)_15__2_, or 45 NANP repeats, [NVDP(NANP)_15__3_, were shown to induce antibodies that bind to the natural CSP on *P. falciparum* sporozoites and to block the invasion of human hepatocytes by the parasite [27,28].

The present report describes an enzyme-linked immunosorbent assay (ELISA) for the evaluation of the immunogenicity of CSP-based *P. falciparum* malaria vaccine candidates, relying on the quantification of human IgG directed against NANP epitopes. R32LR, a recombinant protein consisting of two NVDP oligopeptides and 30 NANP repeats linked to the dipeptide Leu-Arg [NVDP(NANP)_15__2_LR [29,30], was used as coating antigen. The ELISA has been validated according to ICH guidelines [31] and has demonstrated precision, linearity, specificity, robustness, non-interference and stability. Furthermore, a new His-tagged R32LR antigen and a human anti-R32LR monoclonal antibody have been generated, which could extend the operational lifetime of this anti-CSP repeats ELISA.

## Methods

### Coating antigen

#### R32LR

R32LR is a recombinant protein produced in AR58 *Escherichia coli* strain with a temperature induction process and purified by three precipitation steps, followed by reversed-phase high performance liquid chromatography and size-exclusion chromatography, as already described [29]. The final sample buffer was 0.2 M phosphate buffer, pH 6.5. The protein was stored in aliquots at -80°C until use.

#### His-R32LR

His-R32LR was constructed with six histidine residues at the N-terminus. Briefly, a plasmid encoding an *E. coli* codon-optimized R32LR DNA sequence preceded by a histidine-tag was obtained from GENEART AG (Regensburg, Germany). His-R32LR was subcloned in a pET29a plasmid by insertion between *Nde*I and *Sac*I sites, and transformed in the BLR(DE3) *E. coli* strain. Expression of the recombinant protein was obtained by addition of isopropyl β-D-1-thiogalactopyranoside (1 mM) before the temperature was shifted to 39.5°C for 4 h. For purification purpose, bacterial cells were grown in fermenter using fed-batch method and the same induction strategy. The bacterial pellet was suspended in 50 mM phosphate buffer, pH 7.5 containing 300 mM NaCl, 5% glycerol (v/v), 0.1% sodium deoxycholate (w/v), supplemented with complete protease inhibitor cocktail (Roche, 1 tablet/50 ml buffer). The cells were lysed by three French press extractions at 15,000 psi. After refrigerated centrifugation for 30 min at 17,400 × *g*, the supernatant was harvested and filtered through a 0.22-μm membrane.

His-tagged R32LR was further purified through a nickel column (Ni NTA superflow, Qiagen) with elution by imidazole gradient. The pooled positive fractions were dialysed against 50 mM Tris, pH 7.5, containing 5% glycerol (v/v) and subjected to ion-exchange chromatography (Mono-Q column, Pharmacia). The positive fractions were pooled and dialysed against 10 mM phosphate buffer, pH 6.6, containing 150 mM NaCl. R32LR (and His-R32LR) is neither detected by Coomassie blue nor by UV absorbance at 280 nm [29], but high-performance liquid chromatography coupled with detection at 205 nm was used to control batch homogeneity. Protein purity was evaluated by Coomassie blue-stained electrophoresis gel to visualize any contaminating protein, and on western blot membrane probed with rabbit anti-serum targeting *E. coli* lysate proteins. Protein identity was evaluated by western blotting using a murine monoclonal antibody recognizing NANP (in-house; R3G12 antibody). Endotoxin concentration in the final product was determined by Limulus amoebocyte lysate -kinetic QCL assay (Cambrex). R32LR and His-R32LR concentrations were determined by dosage of total nitrogen content. Briefly, after pyrolysis, nitrogen compounds were converted into nitrogen monoxide which then reacted with ozone to produce NO_2*_. NO_2*_ emitted a photon which was detected after signal amplification in an Antek 9000 device (Alytech, France). After purification, both antigens were kept in aliquots at -80°C.

### Serum samples

The assay was developed by using serum samples from healthy subjects in different clinical trials, and taken at various time points after vaccination with RTS,S. RTS,S is composed of a polypeptide chain of 19 NANP repeats and a C-terminal region of *P. falciparum* (3D7) CSP encompassing amino acids 207–395, fused to hepatitis B surface antigen (HBsAg), and an unfused (S) polypeptide of 226 amino acids of HBsAg, spontaneously forming a virus-like particle [4,32,33].

Negative control serum samples were obtained from healthy adults living in malaria non-endemic areas and who were thus considered as anti-CSP repeats antibody seronegative. Positive control samples were pools of post-vaccination serum samples.

As no international standard preparation is available for the determination of anti-CSP repeats antibody concentration in serum, a human sample from the recipient of an exploratory malaria vaccine was taken to constitute the reference standard for the assay, to which a concentration of 109 ELISA units per ml (EU/ml) was attributed (IR2 reference standard, kindly provided by the Walter Reed Army Institute of Research). All study participants and serum donors have given written informed consent for the use of their serum for anti-CSP repeats test development.

### Reference human monoclonal antibody

Hybridomas producing anti-R32LR antibodies were generated using a method described previously [34]. In brief, human peripheral blood mononuclear cells (PBMC) collected from an individual vaccinated with the RTS-S vaccine candidate were injected in the spleens (1-2 × 10^7^ cells per animal) of three SCID mice (C.B-17 Prkdc^scid^/Prkdc^scid^). Six days later the mice were bled and their plasma anti-R32LR antibodies were measured using the in-house ELISA. On day 7, the mouse displaying the highest anti-R32LR concentration was sacrificed, the spleen was removed and a cell suspension was prepared. Human PBMC-SCID spleen cells were mixed with K6H5/B5 heteromyeloma cells at a 5:1 ratio, and polyethylene gly-col 1500 (50% v/v; Boehringer Mannheim, Mannheim, Germany) was added for 2 min before being washed away. Fused cells (5 × 10^4^) were cultured in microtiter plate wells in 200 μl of medium supplemented with human recombinant insulin (10 μg/ml, Boehringer Mannheim), ouabain (1 μM, Sigma, St. Louis, MO), hypoxanthine-aminopterin-thymidine (Life Technologies, Belgium) and 10% v/v BM Condimed HI (Boehringer Mannheim). Cultures were replenished with fresh medium every other day and individual wells were checked for cell growth first and anti-CSP antibody production subsequently. Seven anti-R32LR antibody-producing cultures were selected, subcloned and further expanded.

The seven hybridomas, producing anti-R32LR IgG1, were cultured in CELLine 1000 (Integra Bioscience, Chur, Switzerland), a membrane based disposable cell culture system. For optimal production level the device was inoculated with 50 × 10^6^ cells in Dulbecco’s modified Eagle medium at high glucose concentration supplemented with glutamine, sodium pyruvate, essential and non-essential amino acid, a cocktail of antibiotics and 5% fetal bovine serum. Eighty percent of the production medium and the entire nutrition medium were changed twice a week.

Systems were stopped after 37 days of culture including preculture phase, and 2 hybridomas (MAL 1C and MAL 2A) showed a productivity of 19 mg/month/150 million cells. Monoclonal antibodies were purified by affinity chromatography on Protein A-sepharose Fast Flow (GE Healthcare), aliquoted and stored in phosphate-buffered saline at -20°C before being used as standard in anti-CSP repeats ELISA.

### R32LR ELISA

IgG antibodies directed against the CSP repeat region were measured as follows. R32LR was coated (100 μl/well of a solution of 1.25 μg/ml prepared in 0.05 M carbonate/bicarbonate buffer, pH 9.4-9.8) onto a 96-well polystyrene plate (F96 MaxiSorp, Nunc) for 14-16 h at 5 ± 3°C. After coating, plates were washed 3 times with phosphate-buffered saline (PBS), pH 7.4, supplemented with 0.1% Tween-20 (v/v; Sigma, ref P1379). Nonspecific binding sites were satura-ted with 200 μl/well of blocking buffer, consisting of PBS (pH 7.4) containing 0.1% Tween-20 (v/v) and 0.5% skimmed milk (Becton Dickinson, ref 232100), for 2 h at room temperature (RT) on an orbital shaker. No washing step occurred after blocking but the plate was turned upside down to remove blocking buffer, and gently tapped down on clean blotting paper. Then, eight serial twofold dilutions of standard, controls and samples were added (100 μl/well) and incubated for 2 h at 37°C.

The plates were washed thrice before peroxidase-conjugated anti-human IgG rabbit antibody (Dako, ref PO214) diluted in blocking buffer was added for 30 min incubation at RT. After another washing step, the chromogen substrate mix was added and incubated for 30 min at RT. The chromogen substrate mix was prepared extemporaneously and consisted of four volumes of substrate buffer [Na_2_HPO_4_ (Merck 1.06586), citric acid (Merck 1.002441000) supplemented with 0.006% H_2_O_2_ (v/v) and adjusted to pH 4.1-4.5] mixed with 1 volume of 3,3’,5,5’-tetramethylbenzidine solution (Sigma, ref T-0440). The colorimetric reaction was stopped by the addition of 50 μl of 1 N sulphuric acid, before reading the assay plate at 450 nm in a microtiter plate reader.

An un-weighted 4-parameter logistic (4-PL) fitting algorithm [35] was applied to the standard curve, allowing the determination of antibody concentration in the samples, expressed in ELISA Units per milliliter (EU/ml).

### Anti-hepatitis B antigen ELISA

Anti-HBsAg antibodies were quantified using Abbott’s AxSYM Micro-particle Enzyme ImmunoAssay (MEIA) platform with the dedicated reagent-pack AUSAB, using a standard curve as described by the manufacturer.

### Assay validation

For the validation of the assay, the following parameters were evaluated according to the The International Conference on Harmonisation of Technical Requirements for Registration of Pharmaceuticals for Human Use (ICH) guidelines: precision, linearity, specificity, robustness, interference and stability. Accuracy could not be determined, as no reference analyte of known purity is available. Furthermore, the proposed ELISA could not be compared with another well-characterized gold-standard assay, as no such assay is available.

### Precision

Precision can be defined as the closeness of agreement between independent test results obtained under stipulated conditions, called intermediate precision and repeatability conditions. Intermediate precision expresses the precision obtained under conditions mimicking the routine assay conditions. Repeatability expresses the precision under minimal variable conditions. For the determination of repeatability and intermediate precision in the ELISA, 12 serum samples from RTS,S-vaccinated subjects spanning a large range of anti-CSP repeats antibody concentrations were used, measured in duplicate by three different operators on four different days, equivalent to 288 measurements. For the determination of repeatability, every sample was tested in duplicate on the same plate.

Precision was considered acceptable if it was in line with the descriptive statistics derived from our in-house database compiling repeatability and intermediate precision for 138 ELISAs over the last five years, with application of the same experimental design, that is: the 90^th^ percentile of coefficient of variation (CV) are 12.93% for repeatability and 31.92% for intermediate precision, respectively.

### Linearity

The linearity of an analytical procedure is defined as its ability (within the analytical range) to provide a measurement directly proportional to the analyte concentration. Linearity is confirmed if the degree of underestimation and overestimation does not exceed 20%. Sixty sera from RTS,S-vaccinated subjects, of pre-defined anti-CSP repeats antibody concentration, were used. Sera with a high anti-CSP repeats antibody concentration were pre-diluted in order to obtain a concentration situated in the upper part of the standard curve. All samples were serially diluted twofold in the microtiter plate. The last dilution step for each sample was expected to give an anti-CSP repeats concentration situated in the lower part of the standard curve. Then, anti-CSP repeats antibody concentration in all serially diluted samples was measured according to the assay procedure.

### Specificity

The specificity is the ability to assess unequivocally the analyte in the presence of components that may be expected to be present. Two types of experiments were conducted to assess the specificity of the proposed ELISA. First, the specificity was investigated by testing 14 serum samples from individuals naïve for malaria but vaccinated with HBsAg [36] to demonstrate that the ELISA does not detect any anti-HBsAg IgG. Second, a competition experiment with AMA-1 (*Plasmodium falciparum* apical membrane antigen-1, kindly provided by the Walter Reed Army Institute of Research), HBsAg, and R32LR was conducted. The sera containing specific anti-CSP repeats antibodies were pre-mixed with either of these antigens (final concentration: 10 μg/ml in blocking buffer) and incubated for 24 h at room temperature before being processed further according to the assay procedure.

### Robustness

The robustness of an analytical procedure is the measure of its capacity to remain unaffected by small but deliberate variations in method parameters, which provides indication of its reliability during normal usage. First, three timings for the coating with R32LR were evaluated: 14, 16 and 18 h (standard timing being 16 h). Second, the impact of freeze-thaw cycles from -80°C to room temperature of the coating antigen R32LR was assessed by evaluating the anti-CSP repeats geometric mean concentration and intermediate precision after 1, 2 and 3 freeze-thaw cycles.

### Interference

Interferences are defined as artefactual increase or decrease in apparent concentration of an analyte due to the presence of a substance or a treatment.

#### Analyte stability at various temperatures and impact of freezing/thawing cycles

Ten samples from RTS,S-vaccinated subjects were subjected to the following conditions: overnight storage at -20°C, or 3 cycles (24 h duration each) of freeze-thawing (-20°C to RT), or storage at room temperature for 24 h, before anti-CSP repeats ELISA.

#### Heat-inactivation of the serum samples

The effect of complement-inactivation on the measurement of CSP repeats-specific IgG concentration was assessed on 100 sera (30 negatives, 70 positives). Each of the 100 sera tested was divided equally into two vials, one for control and one for heat-inactivation. Paired samples were tested by the same operator but on different days. Complement-inactivation consisted of incubation at 56°C during 30 minutes.

### Stability

To evaluate the stability of the anti-CSP repeats ELISA, two samples (a low-concentration positive control and a high-concentration positive control, prepared by pooling, homogenizing and aliquoting samples previously demonstrated to be positive) were tested in each plate on every run performed by the laboratory since the start of the routine testing in 2007. The limits of these controls were computed on log_10_-transformed concentrations using a minimum of 40 independent values.

To complete the assessment of the stability of the anti-CSP repeats ELISA, an additional internal blind proficiency panel consisting of 50 samples that have been previously tested has been re-analysed every six months, starting in 2008.

### Determination of assay characteristics

The calculation of the limit of detection (LOD) and the limit of quantification (LOQ) was based on a methodology from The Centers for Diseases Control and Prevention [37]. Each standard curve was fitted using a four parameters logistic (4PL) and the 95% confidence interval (95% CI) was considered. For a standard curve, the LOD is the concentration corresponding to the interpolated intersection of the upper 95% CI of the lower asymptote with the 4-PL fit of standards data. The LOQ is the concentration corresponding to the interpolated intersection of the upper 95% CI of the lower asymptote with the lower 95% CI of the 4-PL fit of standards data. LOD and LOQ were computed for each curve and 95^th^ percentile of LODs and 95^th^ percentile of LOQs were assimilated respectively as the final LOD and the final LOQ.

The cut-off of the ELISA was based on the upper limit of the 99.9% one-sided confidence interval of the anti-CSP repeats response in a naïve malaria-population. The experiment was performed on 108 serum samples obtained from healthy adults living in a non-endemic malaria region, hence assumed not to contain anti-CSP repeats antibody. The log_10_ -transformed optical density (OD) corresponding to the first dilution of each sample were averaged and the standard deviation was determined. In order to get a value in ELISA Units/ml, the upper limit of the one-sided 99.9% confidence interval of the mean was interpolated from the 4PL function built on the average ODs of the standard curves incubated on the 20 plates. Finally this value was multiplied by 50, the minimum dilution factor of the sample.

For the assessment of the analytical range, the lower limit was set at the LOQ and the upper limit was defined as the upper limit of linearity.

### Statistical analysis

Statistical analyses were performed on log_10_-transformed data.

For the validation of assay precision, analysis of variance was performed on the transformed data by using the MIXED procedure of the SAS system, with all factors considered as random. Computations were done on the transformed values but the variability in terms of CV was expressed relative to the non- transformed concentrations.

For linearity, the ratio between successive dilutions was evaluated for each sample. This ratio was estimated by linear regression and computed from the slope of this regression (log (concentration) versus log (dilution)). These ratios were summarized by calculating the 5^th^ percentile, median and 95^th^ percentile.

To evaluate the effect of three freeze-thaw cycles or the effect of 24 h storage at room temperature, analysis of variance was performed by using the MIXED procedure of the SAS system, with the factor condition considered as fixed. The heat-inactivated condition was compared with the non-inactivated condition by means of a paired t-test.

The robustness of the assay was evaluated by analysis of variance using the MIXED procedure of the SAS system, with the factor incubation considered fixed, for the effects of coating time. To study the conditions of storage of the coating antigen, analysis of variance was used (MIXED procedure of the SAS system) with the factor condition considered fixed, followed by one-sided Dunnett test. The effect of sample inactivation was analysed using a concordance analysis, Deming regression [38-40] and correlation analysis.

Stability was evaluated using on the one hand two positive controls and on the other hand a blind proficiency panel of 50 samples. The quality control (QC)-specifications of each of the two controls were defined by computing standard deviation through all data and uncertainty of the mean was taken into account with an alpha risk set at 1% for each of the two positive controls. The stability results using the panel of 50 samples were analysed with a Deming’s regression and a concordance analysis.

## Results

### Assay characteristics

The LOD was estimated at 0.17 EU/ml, rounded off to 0.2 EU/ml and the LOQ was estimated at 0.31 EU/ml rounded off to 0.3 EU/ml.

The cut-off of the ELISA, to allow the distinction between anti-CSP repeats negative and positive samples, was calculated from 108 serum samples from malaria-naïve individuals and estimated at 0.49 EU/ml, rounded off to 0.5 EU/ml.

For the analytical range, the lower limit was set at the limit of quantification, i.e. 0.3 EU/ml. The upper limit corresponded to the upper limit of linearity, found to be 2440 EU/ml (see below).

### Assay validation

#### Precision

To determine the intermediate precision, serum samples taken from 12 RTS,S-vaccinated subjects were tested by different operators on different days. To determine the repeatability, every sample was tested in duplicate on the same plate (each combination of factors is repeated). Results of intermediate precision and repeatability are shown in Table 1. The intermediate precision CV was estimated at 22.46% with a repeatability CV of 4.69%.

**Table 1:** Intermediate precision and repeatability results

**Sample Code**	**Number of observations**	**GMC (EU/ml)**	**CV between repeats (%)**	**CV between operators (%)**	**CV between days (%)**	**CV between days & operators (%)**
1	24	0.9	7.72	12.77	45.10	45.10
2	24	4.9	2.93	9.80	34.75	34.75
3	24	27.0	2.33	9.30	12.32	13.38
8	24	36.7	2.91	9.67	12.12	13.98
4	24	44.8	3.99	10.47	13.72	15.23
5	24	52.0	1.66	9.56	11.11	13.70
10	24	52.5	3.08	12.98	11.79	16.52
7	24	57.2	3.86	12.01	21.20	23.38
6	24	58.6	3.79	12.43	11.73	15.63
9	24	77.2	4.24	13.02	11.81	16.58
12	24	88.9	9.35	18.68	17.56	22.78
11	24	89.5	4.39	13.18	13.64	18.14
ALL	288	32.0	4.69	12.24	20.56	22.46

CV, coefficient of variation; GMC, geometric mean concentration of replicates.

Overall analysis and analysis by sample.

Examination of the data showed that the samples 1 and 2, with the lowest concentrations, gave the highest CV for the assay intermediate precision (respectively 45.10% and 34.75%). To investigate further any potential relationship between low concentration and high CV, a second experiment was conducted using eight samples with low concentrations, i.e. ranging from 1.4 EU/ml to 11.9 EU/ml. The eight samples were assayed in duplicate by three operators; the operators performed the assay on different days (the two factors “operator” and “day” are then confounded for the statistical analysis). Results are shown in Table 2 and demonstrate that low concentrations were measured with a similar precision as high concentrated samples (overall CV = 15% for samples with a low concentration and overall CV = 22.46% for samples displaying a concentration up to 89.5 EU/ml). These precision data are in line with the requirements as defined in the Material and Methods section.

**Table 2:** Repeatability and intermediate precision results on samples with low concentration

**Sample Code**	**Number of observations**	**GMC (EU/ml)**	**CV between repeats (%)**	**CV between days & operators (%)**
1	6	1.4	9.80	27.15
2	6	2.2	0.00	11.49
4	6	4.3	1.45	14.68
6	6	4.6	3.77	5.19
3	6	5.1	1.67	10.25
5	6	6.0	2.40	13.37
7	6	7.0	2.33	10.83
8	6	11.9	3.94	8.33
ALL	48	4.5	4.21	14.86

CV, coefficient of variation; GMC, geometric mean concentration of replicates.

Overall analysis and analysis by sample.

#### Linearity

Sixty sera from RTS,S-vaccinated subjects, covering a broad range of anti-CSP repeats antibody concentration were used. For each sample, the ratio between concentrations corrected for dilution and those corresponding to successive dilutions were estimated. The median, the P5 and the P95 of the 60 computed ratios were respectively 1.002, 0.958 and 1.035.

After three serial dilutions, in 90% of the cases, the degree of underestimation and overestimation were not greater than 12.1% [(1-0.879) x 100] and 10.9% [(1.109-1) x 100], respectively. These data are within the specifications of +/- 20%.

In this respect these results show that the anti-CSP repeats ELISA was linear within the set of dilutions used, corresponding to concentrations from 1.1 EU/ml to 2440 EU/ml.

#### Specificity

Because RTS,S is composed of a *P. falciparum* CSP region coupled to HBsAg, RTS,S vaccination induces high amounts of anti-HBsAg antibodies. Therefore, it was relevant to demonstrate that these antibodies do not interfere in the ELISA. Results are shown in Table 3. Out of 14 samples tested, only one (sample number 5) gave a positive anti-CSP repeats IgG response, but this response was equal to the cut-off (0.5 EU/ml). This was considered as a minor impact considering the imprecision at this level of concentration. Therefore, it could be concluded that anti-HBsAg antibodies do not interfere with the results of the anti-CSP repeats ELISA.

**Table 3:** Specificity of anti-CSP repeats ELISA using hepatitis B-positive, malaria-negative serum samples

**Sample Code**	**Anti-CSP repeats antibody concentration (EU/ml)**	**Anti-HBsAg antibody concentration (mIU/ml)**
1	<0.5	41.5
2	<0.5	22.3
3	<0.5	192.6
4	<0.5	654.2
5	0.5	25.1
6	<0.5	1979.0
7	<0.5	11680.0
8	<0.5	649.5
9	<0.5	5.1
10	<0.5	38.1
11	<0.5	10.7
12	<0.5	138200.0
13	<0.5	509.1
14	<0.5	157.4

CSP, circumsporozoite protein; HBsAg, Hepatitis B surface antigen.

**Figure 1:**
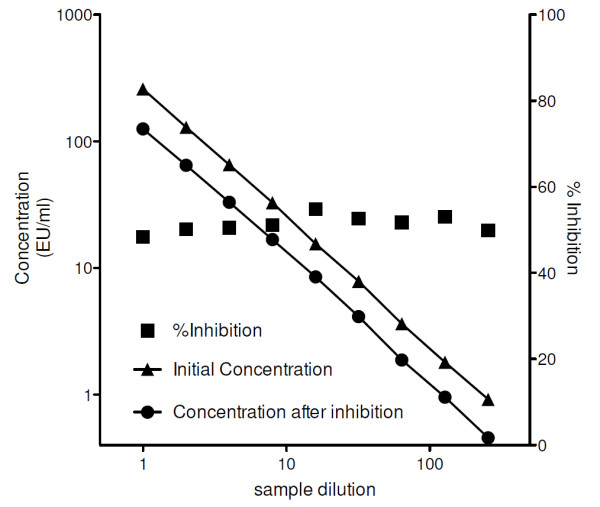
Specificity demonstrated by inhibition of a serially diluted sample.

Further, a competition experiment was performed in which serum samples were pre-incubated with AMA-1, HBsAg, R32LR, or assay diluent (control) before being used in the assay (Table 4). No inhibition was observed with AMA-1 or with HBsAg, whereas a 50% inhibition on average was observed with R32LR, which is a further demonstration of specificity. The similar degree of inhibition (~50%) by the same amount of R32LR (10 μg/ml) in samples of different serial dilutions suggests that this limited level of inhibition is independent of the quantity of antibodies present in the sample (Figure 1).

**Table 4:** Specificity of anti-CSP repeats ELISA using sera pre-incubated with different antigens

**Sample Code**	**Concentration of anti-CSP repeats antibody (EU/ml) Inhibited with:**				**% inhibition**		
	**R32LR**	**AMA-1**	**HBsAg**	CONTROL*	**R32LR**	**AMA-1**	**HBsAg**
1	37	70	69	76	51.3	7.9	9.2
2	80	134	141	138	42.0	2.9	−2.2
3	56	97	102	105	46.7	7.6	2.9
4	97	156	160	162	40.1	3.7	1.2
5	146	249	243	250	41.6	0.4	2.8
6	51	89	98	97	47.4	8.2	−1.0
7	84	148	167	167	49.7	11.4	0.0
8	124	213	223	217	42.9	1.8	−2.8
9	121	222	235	222	45.5	0.0	−5.9
Mean					45.2	4.9	0.5

AMA-1, apical membrane antigen-1; CSP, circumsporozoite protein; HBsAg, hepatitis B surface antigen.

* control: no inhibitory antigen used (co-incubation with diluent).

#### Robustness

The robustness of the assay was evaluated by varying the incubation time for antigen coating (Table 5). Three timings were evaluated: 14, 16 and 18 h, and 16 samples (2 anti-CSP repeats antibody negative and 14 positive) were assayed. The overall *P* value for differences between the three conditions was <0.0001. There was a significant difference (*P* <0.0001; Dunnett’s test) between the standard condition (16 h) and the prolongation by 2 h of the coating incubation time. Indeed, the concentration measurements were 13% lower with 18 h antigen coating, whereas no variation was seen between 14 h and 16 h of antigen coating. Based on these results, the standard coating time was set at 14-16 h.

**Table 5:** Influence of the coating time on IgG concentration

**Sample code**	**Anti-CSP repeats concentration (EU/ml) after coating during:**		
	**14 h**	**16 h**	**18 h**
1	11.3	11.8	10.8
2	7.9	8.5	7.5
3	156.7	174.0	158.2
4	135.7	139.9	118.5
5	642.3	682.9	595.9
6	701.5	635.6	578.5
7	1322.7	1237.7	1047.1
8	1397.4	1320.0	1157.8
9	1779.5	1617.7	1393.3
10	1315.1	1243.2	1082.1
11	1371.9	1298.5	1107.6
12	1180.9	1108.6	974.7
13	1940.2	1923.2	1564.8
14	2124.0	2126.2	1739.9
GMC	468.66	462.31	401.29

CSP, circumsporozoite protein; GMC, geometric mean concentration.

In another series of experiments, the coating antigen was submitted before coating to three freeze-thaw cycles from -80°C to room temperature and kept at room temperature during at least 3 h during each cycle. The anti-CSP repeats ELISA was then performed on a panel of 120 serum samples (30 anti-CSP antibody negatives, 90 positives). It was shown that freeze-thaw cycles and short-term storage at room temperature did not affect the intermediate precision or the LOQ of the test. The observed intermediate precision was estimated at 23.8% and the LOQ was demonstrated at 0.04 EU/ ml. These results are in line with the precision and LOQ described earlier.

#### Interference

In routine procedure, sera may need to be re-tested, hence frozen and thawed several times. It is therefore important to evaluate if the determination of anti-CSP repeats antibody concentrations is affected by several freeze/thaw cycles. The effect of storage at room temperature for 24 h was also assessed (to mimic the long sample handling time that may occur). The samples could be stored at room temperature for up to 24 h and they tolerated three freeze-thawing cycles without being affected for their anti-CSP repeats IgG concentration (analysis of variance with the factor condition considered as fixed factor; *P*=0.92) (Table 6).

**Table 6:** Influence of 3 freeze-thaw cycles or storage at room temperature of the serum sample on anti-CSP repeats antibody concentration

**Sample code**	**Concentration (EU/ml)**		
	**Basis condition**	**3 cycles**	**RT**
1	80.7	74.2	74.5
2	122.2	150.5	139.9
3	92.7	93.0	99.5
4	80.6	80.7	77.4
5	57.2	50.0	68.9
6	68.3	72.4	64.2
7	94.8	89.3	87.0
8	111.0	118.5	112.0
9	41.5	45.2	41.7
10	610.0	596.7	591.7
GMC	97.37	98.57	98.43
GMR, compared with basis condition (95%CI)		1.01 (0.94-1.09)	1.01 (0.94-1.08)

GMR, geometric mean of the individual ratios.

**Figure 2:**
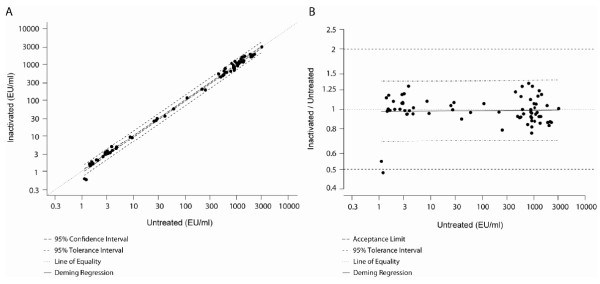
**Comparison of heat inactivated samples with non-inactivated samples. A**, Deming regression (ultrastructural model) of non-inactivated serum (untreated‘) as a function of inactivated serum samples (inactivated) where variances are considered as equal. **B**, Plot of the bias - Deming regression represented with ratios (inactivated/untreated) versus inactivated (ultrastructural model) where variances are considered as equal.

In routine procedure, the complement was not inactivated in the samples. However, it may happen that sera previously complement-inactivated for other analyses are subsequently used in the anti-CSP repeats ELISA. Therefore, it was necessary to ensure that this inactivation had no impact on the concentration of CSP repeats-specific IgG. The results showed that complement inactivation did not affect the ability of the test to correctly discriminate positive from negative samples, demonstrating 100% (95% CI: 96.38 – 100.00) agreement; concentrations remained equivalent based on the geometric mean of the individual ratios (1.01 [95% CI 0.97-1.06]) and on the outcome of the Deming regression (slope 0.9981 [95% CI: 0.9837 – 1.0128], correlation r = 0.9982) (see Figure 2).

#### Stability

**Figure 3:**
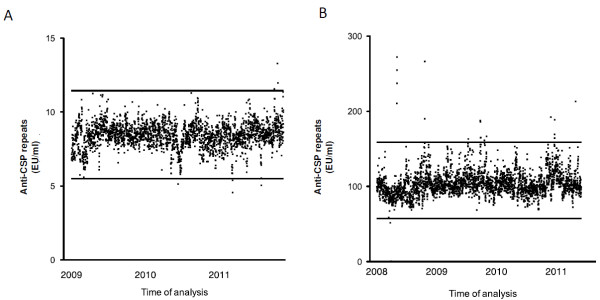
**Stability of quality control samples.A**, Stability of the Low Titer Quality control sample measured in each plate of the ELISA run (with the lower and upper rejection limit). **B**, Stability of the High Titer Quality control sample measured in each plate of the ELISA run (with the lower and upper rejection limit).

To assess the stability of the assay, the same low and high concentration control samples were tested in each plate of the anti-CSP repeats ELISA. Results are illustrated in Figure 3, and it was shown that values were within QC-specification in 99.81% and 99.34% of cases for the low and high concentration samples, respectively.

Stability of the ELISA was also demonstrated by using a blind proficiency panel re-analysed every 6 months (Table 7).

**Table 7:** Summary of the results obtained with the same serum panel tested every 6 months

	**Time point 1**	**Time point 2**	**Time point 3**	**Time point 4**
Slope of the Deming's regression^a^	0,99 (0,98-1,00)	1,00 (0,98-1,01)	0,99 (0,97 - 1,01)	1,01 (1,00 - 1,03)
Bias of the Deming's regression^b^	OK	OK	OK	OK
GMR^c^	1,140	1,015	1,027	1,03
Agreement (%)^d^	100	100	100	98
McNemar p-value^e^	OK	OK	OK	1,000

^a^Slope must be equivalent to 1.

^b^Bias must be between 0.5 and 2.00 for the whole range.

^c^GMR must be between 0.80 and 1.25.

^d^Agreement must be >80%.

^e^McNemar value must be ≥0,05.

GMR, geometric mean of the individual ratios.

### Production and bridging of His-R32LR

Four hundred milligrams of His-R32LR were obtained from 30 g of bacterial pellet. After purification by nickel affinity and ion exchange chromatography, the purity of His-R32LR was indirectly demonstrated by the fact that no band was stained by Coomassie blue after gel electrophoresis. In addition, no band was detected by western blotting, using antibody directed against total *E. coli* proteins. The expected multiband pattern, corresponding to the profile of the original R32LR, was seen after western blotting using R3G12 antibody, confirming protein identity. Batch size homogeneity was confirmed by high-performance liquid chromatography, showing a single peak at 205 nm, and final protein concentration was determined by dosage of total nitrogen content. Endotoxin concentration was 27.5 EU/ml.

**Figure 4:**
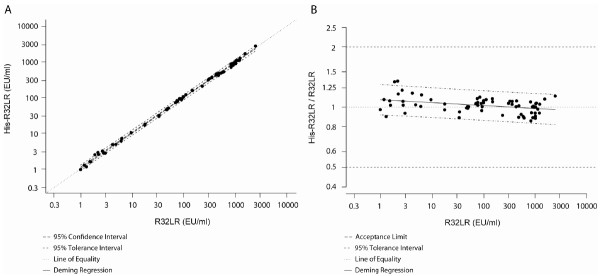
**Comparison of His-R32LR with the original R32LR as coating antigen.A**, Deming regression (ultrastructural model) of His-R32LR as a function of R32LR where variances are considered as equal. **B**, Plot of the bias - Deming regression represented with ratios (His-R32LR/R32LR) versus R32LR (ultrastructural model) where variances are considered as equal.

Sample concentrations were equivalent when measured by the anti-CSP repeats ELISA with either His-R32LR or the initially used R32LR as coating antigen, which is demonstrated by the geometric mean of the individual ratios being 1.02 (95% CI: 1.00-1.04), and the slope and correlation of the Deming’s regression (Figure 4) being respectively 0.9854 (95% CI: 0.9766-0.9942) and 0.9993. The positive/negative agreement (Table 8) was very high [98.99% (95% CI: 94.50-99.97%) agreement and Mc Nemar p-value=1,000] with only one sample returning as false positive result using His-R32LR. However, the reported concentration of this sample (0.517 EU/ml) was close to the assay’s cut-off (0.5 EU/ml). It is important to note that during routine testing, extremely low titer results (0.5 – 1 EU/ml) are subjected to a confirmation (re-testing) procedure.

**Table 8:** Contingency table showing the number of negative and positive samples depending on the coating antigen, R32LR or His-R32LR

		**His-R32LR**		
		**Negative**	**Positive**	**Total**
R32LR	Negative	29	1	30
	Positive	0	69	69
	Total	29	70	99

### Bridging of monoclonal antibody with the ELISA standard

**Figure 5:**
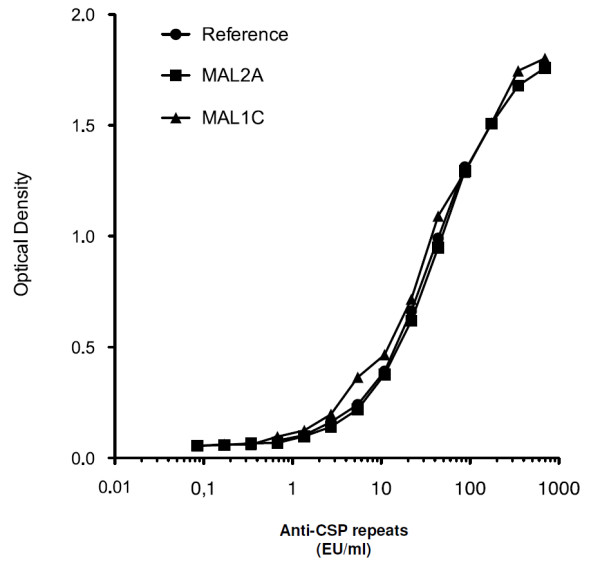
Optical density of serial dilutions of the original standard serum obtained from an exploratory malaria vaccine recipient, compared to the monoclonal MAL2A antibody and the monoclonal MAL1C antibody.

The human monoclonal antibodies (MAL 1C and MAL 2A) were tested in the anti-CSP repeats ELISA against the internal reference in twofold serial dilutions from 1/50 down to 1/1.34 × 10^10^. Sigmoidal dose range response curves were obtained for both monoclonal antibodies and the curves were superimposable to that of the originally used standard (Figure 5). The obtained OD values within the analytical range of the ELISA were used to determine that MAL 1C and MAL 2A represent 11,130.0 EU/ml and 16,372.9 EU/ml of anti-CSP repeats antibody concentration, respectively. Accordingly, a value of 500 EU/ml in the current standardization was found equivalent to 16 μg/ml and 18 μg/ml for MAL1C and MAL2A, respectively. The variability observed between the different individual concentrations from each dilution used to calculate the concentration was limited (MAL 1C inter-dilution % CV = 4.9 and MAL 2A inter-dilution %CV = 0.5).

Considering that both preparations displayed antibody concentrations 100- to 150-fold higher than the actually used standard and low amount of standard is needed to perform the anti-CSP repeats ELISA, 1 ml of one of these monoclonal antibody preparations will allow the quantification of up to 2.5 million clinical trial samples.

## Discussion

An R32LR-based ELISA that can be used as a standard anti-CSP antibody assay for clinical malaria studies involving CSP-based *P*. *falciparum* vaccine candidates has been developed and validated. This assay fulfills the ICH criteria of precision, linearity, stability and robustness that are adopted by the regulatory authorities for assay validation [41,42]. The critical validation parameters are summarized in Table 9. Panels of blinded serum samples with high and low anti-CSP repeats concentration have been included in the routine testing since the introduction of the assay in 2007 [43], and have confirmed that the test performs within established criteria. In addition, it has been shown that R32LR coating antigen is stable and unaffected by several freeze-thaw cycles. Finally, the operational lifetime of this ELISA is supported by the development of alternative laboratory-based sources for the preparation of key reagents.

**Table 9:** Critical assay parameters

LOD	0.2 EU/ml
LOQ	0.3 EU/ml
Cut-off	0.5 EU/ml
Precision (CV)	22.46%
Linearity	1.1 - 2440 EU/ml
Analytical range	0.3 - 2440 EU/ml

LOD, limit of detection.

LOQ, limit of quantification.

CV, coefficient of variation.

Measuring vaccine immunogenicity with a unique validated immunoassay would allow quantitative comparisons between *P. falciparum* CSP-based vaccine candidates. The anti-CSP repeat region ELISA described here is a relevant choice for this purpose. Being the reference assay for vaccine immunogenicity during the clinical development of the RTS,S vaccine candidate [12,19,43-52], it has an extensive track record of use in adults as well as in the pediatric population. More than 20,000 serum samples from children living in malaria-endemic areas have been evaluated to date with this assay. Using a validated immunoassay would also help to identify the minimum anti-CSP repeats antibody concentration associated with protection. In this respect, attempts to define correlates of protection have already been made with the ELISA presented here in the context of a malaria field trial [19]. One limitation of this assay might be the absence of the N-and C-terminal parts of CSP in the recombinant molecule, implying no detection of antibodies against potential N- and C-terminal epitopes. However, to date, the only antibodies shown to neutralize infectivity of sporozoites are those directed against CSP repeats [22,53], and it is commonly accepted that the central repeat region of CSP is a major immunodominant epitope [54,55]. This suggests that the levels of antibodies against the repeat region may represent a major part of the global anti-CSP response. One may also argue that the ELISA presented here does not give indication on the functionality of the antibodies that are measured. Nevertheless, anti-CSP antibodies are believed to be functional, as has been reported previously [56-60].

Different multiples of NANP-repeats have been used in immunoassays, from (NANP)_3_ to (NANP)_40_[23-26]. Although anti-CSP repeats antibodies can be detected using repeats of different multiples, it has been observed in a comparative analysis that results of higher multiples of NANP-repeats better correlated with each other than with those of lower multiples and that R32LR-based and (NANP)_40_-based ELISAs had higher sensitivity than (NANP)_3_-based ELISA [24]. Such discrepancy may originate from differences in the coating capacity of short peptides compared to longer ones. Also, the number of epitopes on low multiple NANP repeats may be limited and essentially of the linear type, whereas additional conformational epitopes may arise from the folding of high multiple NANP repeat peptides. This has to be emphasized, since the importance of the three-dimensional structure of the CSP repeats for antibody recognition has already been observed [61]. In line with this, a preliminary comparison of R32LR to short (NANP)_3-6_ peptide ELISA did not yield consistent results for the evaluation of anti-CSP repeats antibody concentration with the ELISA described here, while NANP_10_ showed better results (unpublished data). Therefore, an assay with a longer molecule may be superior to uncover the polyclonal antibody repertoire induced by CSP vaccination. In this regard, R32LR may have an extra advantage compared with a long repeat molecule such as (NANP)_40_, since it contains the NVDP sequence in addition to the NANP repeats (as native CSP does), which may also represent a possible extra epitope [24]. Furthermore, in contrast with R32LR, short NANP sequences have never been evaluated in the context of clinical trials.

The advantages of the R32LR-based ELISA have long been challenged by the limited availability of its key component, R32LR, and by the fact that the reference serum derived from an adult RTS,S recipient is a reagent in limited amount. To address this issue, a histidine-tagged R32LR recombinant protein, easy to purify and available in large amounts, was produced and shown to be equivalent to R32LR for the use in the anti-CSP repeats ELISA. In addition, a human monoclonal antibody has been developed that recognizes epitopes on R32LR. This anti-R32LR monoclonal antibody will be a useful standard reagent since it is equivalent to the human anti-CSP positive serum and can be produced in large amounts. Ultimately, it may serve as an international standard preparation.

## Conclusions

An anti-CSP repeats ELISA for the evaluation of the immunogenicity of CSP-based *P. falciparum* vaccine candidates has been validated. Critical reagents have been developed to ensure the continuous application of this assay as a standard anti-CSP ELISA. Establishing a standard immunoassay for the comparison of immune responses to the various malaria vaccine candidates was a main goal in the malaria vaccine technology roadmap [62]. The current report is a step in this direction.

## Abbreviations

AMA-1: *Plasmodium falciparum* apical membrane antigen-1; CI: Confidence interval; CSP: Circumsporozoite protein; CV: Coefficient of variation; ELISA: Enzyme-linked immunosorbent assay; HBsAg: Hepatitis B surface antigen; LOD: Limit of detection; LOQ: Limit of quantification; OD: Optical density; PBMC: Human peripheral blood mononuclear cells; PBS: Phosphate-buffered saline; QC: Quality control; RT: Room temperature.

## Competing interests

The study was funded by GlaxoSmithKline Biologicals SA (GSK). VD, MD, CS, MAD, EJ, JC and PC are employees of GSK. JC, MAD and PC own shares and/or options to shares in GSK. In addition, JC is a designated inventor on patented malaria vaccines, but does not hold a patent for a malaria vaccine. GLR has received fees from GSK for consultancy and lectures on different vaccine topics, and for travel and accommodations for the participation to a congress. The other authors declare no potential conflict of interest.

## Authors’ contributions

EJ, FC, GLR, JC and MAD were involved in the design of the study. CS, EJ, EVB, FC, GLR, ID, MAD, PC contributed to the development and/or review of the study protocol. FC, VD and MD made substantial contribution to the method selection and development. EVB, GLR, PC recruited subjects for clinical trials. EVB, FC and GLR participated to the acquisition of data. CS, EJ, EVB, FC, GLR, ID, JC, MAD, PC analysed and interpreted the results. All authors contributed to the development of the manuscript, were involved in finalizing the manuscript, read and approve the final version.
